# A Single-Subject Method to Detect Pathways Enriched With Alternatively Spliced Genes

**DOI:** 10.3389/fgene.2019.00414

**Published:** 2019-05-09

**Authors:** Alfred Grant Schissler, Dillon Aberasturi, Colleen Kenost, Yves A. Lussier

**Affiliations:** ^1^Department of Mathematics and Statistics, University of Nevada, Reno, Reno, NV, United States; ^2^Center for Biomedical Informatics and Biostatistics, The University of Arizona, Tucson, AZ, United States; ^3^Department of Medicine, The University of Arizona, Tucson, AZ, United States; ^4^The Graduate Interdisciplinary Program in Statistics, The University of Arizona, Tucson, AZ, United States; ^5^BIO5 Institute, The University of Arizona, Tucson, AZ, United States; ^6^Cancer Center, The University of Arizona, Tucson, AZ, United States; ^7^University of Arizona Health Sciences, The University of Arizona, Tucson, AZ, United States

**Keywords:** RNA-Seq, precision medicine, isoform, alternative splicing, systems biology, pathways, local false discovery rate, Hellinger distance

## Abstract

RNA-Sequencing data offers an opportunity to enable precision medicine, but most methods rely on gene expression alone. To date, no methodology exists to identify and interpret alternative splicing patterns within pathways for an individual patient. This study develops methodology and conducts computational experiments to test the hypothesis that pathway aggregation of subject-specific alternatively spliced genes (ASGs) can inform upon disease mechanisms and predict survival. We propose the N-of-1-*pathways* Alternatively Spliced (N1PAS) method that takes an individual patient’s paired-sample RNA-Seq isoform expression data (e.g., tumor vs. non-tumor, before-treatment vs. during-therapy) and pathway annotations as inputs. N1PAS quantifies the degree of alternative splicing via Hellinger distances followed by two-stage clustering to determine pathway enrichment. We provide a clinically relevant “odds ratio” along with statistical significance to quantify pathway enrichment. We validate our method in clinical samples and find that our method selects relevant pathways (*p* < 0.05 in 4/6 data sets). Extensive Monte Carlo studies show N1PAS powerfully detects pathway enrichment of ASGs while adequately controlling false discovery rates. Importantly, our studies also unveil highly heterogeneous single-subject alternative splicing patterns that cohort-based approaches overlook. Finally, we apply our patient-specific results to predict cancer survival (FDR < 20%) while providing diagnostics in pursuit of translating transcriptome data into clinically actionable information. Software available at https://github.com/grizant/n1pas/tree/master.

## Introduction

RNA-Sequencing (RNA-Seq) data offers an opportunity to enable precision medicine, but most methods rely on gene expression alone (Sørlie et al., 2001; Weigelt et al., 2005; Peppercorn et al., 2008; Bastien et al., 2012; Prat et al., 2014). RNA-Seq, however, provides even greater resolution, including messenger RNA (mRNA) diversity for the same protein-coding genomic region – corresponding to distinct *protein isoforms*, created by *alternative splicing* of exons. Most RNA-Seq analytics ignore alternative splicing patterns despite recent evidence that alternative splicing is implicated in nearly a third of common diseases. In cancer, a tumor often displays dysregulation of the cellular machinery that controls alternative splicing (Yoshida et al., 2011; Kaida et al., 2012; Ladomery, 2013; Forootan et al., 2016). Yet, the clinical interpretation of alternative splicing patterns lies largely unexplored.

This study develops methodology and conducts computational experiments to test the hypothesis that pathway aggregation of subject-specific alternatively spliced genes (ASGs) can inform upon disease mechanisms and predict survival, thereby providing clinical interpretation of alternative splicing patterns. By “alternative splicing” patterns, we mean that the distribution of isoforms of a certain gene differs between two samples. Specifically, in the context of cancer, our hypothesis is driven by the high likelihood that comparing non-cancer (“normal”) tissue to cancer tissue will unveil cell-type specific expression in cell-type specific pathways. This, in turn, will affect the proportion of the ASGs in the overall comparison between tissues (where the cell-type elements have changed) and will distribute in pathways. Two facts taken together form this opinion: (1) cellular-specific splicing occurs and (2) if a differentially expressed gene (DEG) occurs between paired samples, it is in part due to the change in activated pathways within the concordant cell types that have become cancerous, and in part due to the change of cell-type proportions in the cancer tissue vs. normal tissue (e.g., the stroma may contain more immune cells that were previously absent).

We and others have recently developed methodological frameworks to clinically interpret individualized signals from molecular data (Chen et al., 2012; Yang et al., 2012; Ahn et al., 2014). In particular, we introduced a statistical framework, N-of-1-*pathways*, to provide subject-specific interpretations of the transcriptome (Gardeux et al., 2014; Li et al., 2017a,b, Schissler et al., 2015, 2018). The methodology focuses on quantifying an individual’s dynamic transcriptional response within cellular pathways, along with providing uncertainty quantification for these metrics. To this end, paired samples (e.g., normal/tumor, before, and after treatment) are obtained from a patient, and gene set analysis (Subramanian et al., 2005; Goeman and Bühlmann, 2007; Khatri et al., 2012) is conducted for the individual without the requirement of large cohorts.

In this study, we propose a novel methodology to improve the clinical interpretation of subject-specific alternative splicing patterns derived from paired RNA-Seq samples. The N-of-1-*pathways* Alternatively Spliced (N1PAS) method transforms a patient’s paired-sample RNA-Seq isoform expression data (e.g., tumor vs. non-tumor, before-treatment vs. during-therapy) into a pathway enrichment profile of ASGs. N1PAS quantifies the degree of alternative splicing using gene-wise Hellinger distances followed by two-stage clustering to determine pathway enrichment using a robust, existing procedure testing procedure – local false discovery rate (locFDR). The single-subject output provides an interpretable *odds ratios* describing the overrepresentation of ASGs along with uncertainty quantification through locFDR.

The article continues with some brief mathematical background and description of the proposed method. Then, several computational experiments explore and validate our proposed methods in clinical samples. In this proof of concept study, we demonstrate the potential for alternative splicing interpretation as one of the Omics signals which should be considered for predicting cancer survival. We also compare the proposed method with alternative approaches. Finally, we conduct extensive simulation studies to explore empirical operating characteristics of N1PAS. A discussion concludes the article.

## Mathematical Background

This section motivates the use of and describes two mathematical concepts employed in the proposed method.

### Hellinger Distance

Our method quantifies alternating splicing between a pair of samples using the *Hellinger distance*. Such an approach has been shown to be useful in the quantification of alternative splicing in the context of clustering (Johnson and Purdom, 2017). Let the estimates of isoform (mRNA) expression for sample A be denoted as *x_gA1_*,…, *x_gAK_g__*, for the K_g_ distinct isoforms annotated to gene g. We define the relative isoform usage as the vector of relative proportions of each isoform, $PgA=\left(\frac{x_{gA1}}{\sum_{k=1}^{Kg}x_{gAk}},...,\frac{x_{gAK}{}_{{}_{g}}}{\sum_{k=1}^{Kg}x_{gAk}}\right)$. Where $\sum_{k=1}^{Kg}x_{gAk}$ is the total gene expression (summed over all isoforms) for gene g. The Hellinger distance between two proportions derived from samples A and B within gene g is given by:

(1) $$H_{g}(p_{gA},p_{gB})-\frac{1}{\sqrt{2}}\sum_{k=1}^{K_{g}}{\left(\sqrt{\frac{x_{gAk}}{\sum_{k=1}^{K_{g}}x_{gAk}}}-\sqrt{\frac{x_{gBk}}{\sum_{k=1}^{K_{g}}x_{gBk}}}\right)}^{2}$$

Simply stated, the *Hellinger distance quantifies dissimilarity between the two distributions of proportions*. The result is a real number H_g_ that resides in the unit interval, with 0 indicating perfect agreement in isoform usage and values tending to 1 indicating an increasing difference in relative isoform distribution of the two samples. Notably, a DEG can also been alternatively spliced by still displaying a large Hellinger distance. Also, the Hellinger distance is symmetric (Equation 1) by definition. To establish a convention, if a gene is not expressed in both samples, we choose to record a missing value for the distance.

### Local False Discovery Rates Through Mixture Modeling

Efron’s local false discovery rates provides a flexible and robust tool for multiple hypothesis testing under correlated test statistics or effect sizes (Efron, 2004, 2007, 2013). RNA-seq data quantifying gene and isoform expression are correlated, both due to the nature of the counting process and biological considerations. Most statistics (including *p-*values) derived from these measurements will also be correlated. So, we need a model that either specifically accounts for this co-expression or does not assume independence. Efron discusses the statistical issues in detail in Efron (2007), including the close relationship of locFDR to other false discovery rates, such as Benjamini–Hochberg (Benjamini and Hochberg, 1995).

Local FDR results from modeling test statistics as arising from a two-component mixture density. Formally, let z_i_ be an observed test statistic from *i* = 1,…, *N* testing procedures. *N* must be large to ensure quality locFDR estimates, say at least in the hundreds. But the z_i_ need not be independent. We assume that the *N*-values can be sorted into two classes (“null” and “non-null”), occurring with prior probabilities of *p*_0_ or *p*_1_ = 1 - *p*_0_:

$$\begin{array}{c} p_{0}=\mathrm{Pr}\{null\}.f_{0}(z)\;density\;if\;null\\ p_{1}=\mathrm{Pr}\{nonnull\},f_{1}\;(z)\;density\;otherwise \end{array}$$

Define the *null subdensity* as:

$$f_{0}^{+}(z)=p_{0}f_{0}(z)$$

And the *mixture density*:

$$f\;(z)=p_{0}f_{0}\;(z)+p_{1}f_{1}\;(z)$$

Then, define the local false discovery rate (locFDR; Equation 2) as the Bayes posterior probability that a case is null given z:

(2) $$fdr\;(z)=\mathrm{Pr}[null|z]=\frac{p_{0}f_{0}\;(z)}{f\;(z)}=f_{0}^{+}\;(z)/f\;(z)$$

The definition provides a straightforward interpretation: It is the probability an observed value came from the null density. In practice, Efron indicates that a locFDR < 0.2 provides strong statistical evidence that the case is from the non-null distribution.

### Methodology: N-of-1-Pathways Alternatively Spliced (N1PAS)

**FIGURE 1:**
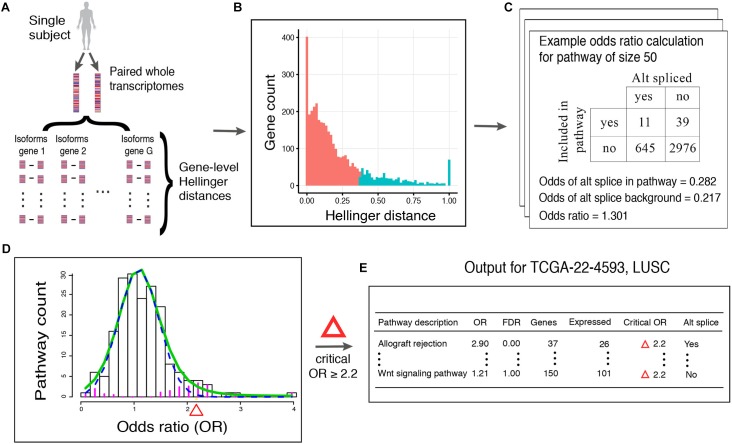
Workflow of N1PAS. **(A)** Isoform-specific mRNA-Seq data are obtained from an individual. Gene-level distances between the two samples indicates the magnitude of alternative splicing. **(B)** Gene-level distances are aggregated across the whole transcriptome and unsupervised clustering classifies genes as alternatively spliced genes (ASGs; blue) and not (red). The vertical axis shows the count of genes with Hellinger distances binned together to form the histogram. Note that since this is a univariate setting, 2-means simply finds a threshold Hellinger distance to classify the genes into two groups. Gene set (pathway) enrichment analysis is conducted by first **(C)** computing the odds ratio (OR) to quantify the relative abundance of ASGs in the pathway vs. genes not in the pathway (background). ORs are calculated for each pathway in the data-base to produce an empirical, subject-specific distribution **(D)**. A local false discovery procedure provides uncertainty quantification (FDR) and classified pathways as enriched using a simple threshold at FDR < 20%. **(E)** Results are tabulated to provide an individualized profile of alternative spliced enrichment.

Here, we describe our proposed method, N1PAS. The approach aims to transform a single subject’s paired transcriptome data into an interpretable, mechanism-based profile of alternatively splicing patterns (Figure 1). We begin by computing a Hellinger distance for each gene (Equation 1) to quantify differential isoform usage between the paired samples (Figure 1A). Once isoform data are transformed into gene-level distances, we then classify genes as either alternatively spliced vs. not using conventional 2-means (as in *k*-means) clustering (Figure 1B). Next, we quantify an enrichment of ASGs within a gene set (*pathway*). That is, odds ratios (OR; Equation 3; Figure 1C) compare the relative abundance of ASGs within the pathway vs. the genes not in the pathway:

(3) $$OR_{\text{pathway}}=\frac{\#of\;ASGs\;in\;pathway/{}_{\#of\;non-ASGs\;in\;pathway}}{\#of\;ASGs\;not\;in\;pathway/{}_{\#of\;non-ASGs\;notin\;pathway}}$$

Then, we calculate locFDR values (Equation 2) by fitting the two-component mixture model to the distribution of pathway odd ratios (Figure 1D). This whole process results in a single-subject, mechanistic profile of alternative splicing, along with effect size and statistical significance (Figure 1E).

## Methods for Computational Experiments

This section describes computational experiments to explore, validate, and apply N1PAS. We conduct these studies using RNA-Seq data derived from clinical samples housed in The Cancer Genome Atlas (TCGA). Pathway annotations are based on the Kyoto Encyclopedia Genes and Genomes (KEGG; Kanehisa and Goto, 2000). Survival data were also retrieved from the TCGA.

### Data Set Acquisition, Preprocessing, Pathway Ontology

Data sets were selected based on the availability of: (1) paired normal-tumor isoform-level quantification from each cancer patient, (2) a KEGG pathway annotated to the same cancer, and (3) survival data. Six TCGA data sets were identified (Table 1) meeting that criteria. The Broad GDAC Firehose was employed to retrieve RNA-Seq data in the form of RSEM normalized isoform expression (downloaded 25/7/2017)^1^. The UCSC Table Browser (Goldman et al., 2015) was used to map isoform identifiers to the corresponding HGNC host gene symbol. In total, 73,599 isoform measurements were associated with 29,181 unique gene symbols. Since for many genes, it is non-trivial to estimate isoform levels correctly due to ambiguity in assigning reads, we used the RSEM adjusted expression values.

**Table 1:** Characteristics from six TCGA RNA-Seq data sets with paired normal-tumor data, survival data, and associated target KEGG pathway.

TCGA	Cancer	Target KEGG pathway description	Number of patients	Number deceased	Isoforms measured
LUSC	Lung squamous cell carcinoma	Non-small cell lung cancer	51	32	73,599
LUAD	Lung adenocarcinoma	Non-small cell lung cancer	58	26	73,599
PRAD	Prostate adenocarcinoma	Prostate cancer	52	0	73,599
THCA	Thyroid carcinoma	Thyroid cancer	59	4	73,599
UCEC	Uterine corpus endometrioid carcinoma	Endometrial cancer	7	2	73,599
BLCA	Bladder carcinoma	Bladder cancer	19	11	73,599

The RNA-Seq data sets were filtered to include patients with paired normal-tumor data. Such patients were identified via the R library TCGA2STAT (Wan et al., 2015). Further, clinical information including survival data for the subjects was queried using this library. The data were normalized using transcripts per million (TPM) to make library size adjusted comparisons between samples derived from the same patient.

Genes were annotated to KEGG gene sets (pathways) using the Bioconductor database KEGG.db version 2.3.5, downloaded 16 Sep 2009. In total, 230 gene sets were downloaded. To improve efficiency of the algorithms developed, the 73,599 isoforms measured were filtered to only those that mapped to a gene annotated to a KEGG pathway (5,879 unique genes), resulting in 18,823 isoform-level quantities for the 5,757 genes measured in the TCGA data set. Following standard practice in alternative splicing analytics (Johnson and Purdom, 2017) only genes with at least 2 and no more than 30 isoforms were retained, leaving 17,088 isoform measurements on 4,133 genes. Lastly, in order to maintain interpretability and stability, the pathways were filtered to have at least 15 and no more than 500 genes – resulting in 206 pathways considered.

### KEGG Target Pathway Validation Study

We aim to validate our methods by exploring N1PAS results within KEGG *target pathways*. Our strategy here was inspired by the work of Diaz et al. (2017). Similar to Diaz et al. (2017), we define a *target pathway* as a KEGG pathway whose description matches the disease associated with the RNA-Seq data set (see Table 1). The validation study will use descriptive statistics and empirical (permutation-based) significance assessments to determine to what extent target pathways were identified as enriched. Differing from Diaz et al. (2017), the analysis is conducted for each individual patient, and, thus patient heterogeneity within target pathways will also be studied. Moreover, we broaden the concept of target pathways to *cancer pathways*, as defined by any KEGG pathway with *cancer* contained in the description. In total, there are nine cancer pathways, five target pathways listed in Table 1 and four additional KEGG pathways, described as *pathways in cancer*, *small cell lung cancer*, *pancreatic cancer*, and *colorectal cancer*. To form descriptive statistics, a *pathway capture rate* is defined as the proportion of patients that found the pathway significantly enriched at locFDR < 20% and odds ratio > 1. In the case of *cancer pathways*, a *cancer pathway capture rate* is the proportion of patients that found at least one of the cancer pathways enriched. Note that N1PAS was carried out using all 206 KEGG pathways and the detection rate of target and cancer pathways was explored to validate our methodology.

The above-mentioned empirical significance assessment entails producing 2000 *null* binary matrices of size *N (patients) × P (pathways)*. The assessment begins with the original matrix of alternatively splicing calls – 1 indicating that a pathway had both an odds ratio greater than 1 and locFDR was less than 0.20, and 0 otherwise. Then each patient’s values (rows) are shuffled. This procedure will preserve the number of 0’s and 1’s within each patient while disrupting the correspondence across individuals and pathways. Thus, each column forms a synthetic *null pathway* with values not tied to any particular pathway annotation while preserving the patient distributional characteristics. Once shuffled, a null pathway is randomly selected and the number of 1’s (significant pathways) are counted to calculate the null capture rate. The procedure is repeated 2000 times to form an approximate null distribution for the capture rate for a single pathway. An *empirical p-value* is the proportion of null pathways with a higher capture rate than the observed target capture rate for a given data set. To assess the capture rate for at least one of nine selected pathways (mimicking the cancer-annotated capture rate), the capture rate of at least one of nine pathways is computed by selecting nine columns at random, without replacement, and counting a “success” as at least one “1” in the vector of 9 values. This procedure is repeated 2000 to produce a null distribution corresponding to the chance of capturing at least 1 out of 9 cancer-annotated pathways. The empirical *p*-value for this assessment is proportion of times the null capture rate is larger than the observed cancer capture rate. The assessment is limited as it makes no adjustment for the fact that smaller pathways correspond to more variable odds ratios (and therefore different probabilities of alternative splicing calls).

There are caveats to using local FDR for testing pathways in the KEGG database. The locFDR implementation in *R* (*locfdr*) contains default parameters that assume there are a large number of tests (at least 1000). Since there are approximately 200 pathways under consideration, custom configurations needed to be developed. Based on practical experience with the pathway odds ratio distributions, the model fits reasonably well using the following heuristic procedures: (1) filter outlier odds ratios and set FDR_loc_ to 0 for those pathways, (2) the number of breaks in the histogram is set to 25, (3) *nulltype* is set to “Central Matching” to provide less conservative results (to compensate for small sample issues), and (4) the mixture density estimate’s degrees of freedom is set to 4. In larger ontologies, program defaults should be adequate, but the model fit should always be inspected. It is important to note that the number of genes in each pathway does not play into any locFDR calculations. We chose to forgo any formal inferences at the gene level as it is rate prohibitive to manually inspect and adjust the fit at the first stage of clustering (Figure 1B). Instead, we employ a 2-means strategy to classify genes as ASGs.

### Disease Subtyping Pipeline Using N1PAS Single-Subject Metrics

**FIGURE 2:**
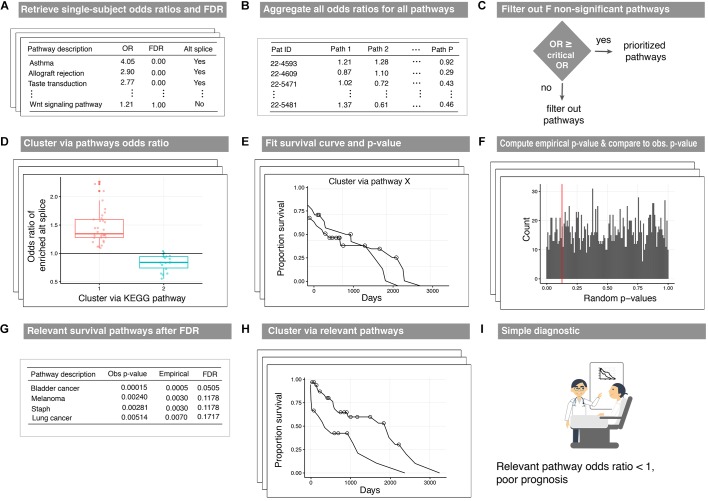
Workflow of the disease-subtyping pipeline. Single-subject N1PAS pathway-level metrics **(A)** are aggregated for the patients in the cohort **(B)**. Then, the dimension is reduced by filtering out pathways that are not significantly enriched in any patient **(C)**. For each prioritized pathway remaining, patients are clustered (unsupervised) into two subtypes **(D)**. Two Kaplan-Meier survival curve estimates are produced along with a log-rank *p*-value **(E)**. An empirical distribution of *p*-values is computed to assess significance **(F)**. The top-prioritized pathways are called survival relevant pathways **(G)**. **(H)** displays the survival curves for patient clusters derived in relevant pathways. The curves presented here depict the results from a clustering that passed the empirical *p*-value threshold from **(E)**. There is no difference in the clustering approach – just an illustration of a successfully identified survival-relevant pathway. Lastly, decision-friendly cutoffs are provided to inform at the point of care **(I)**.

We now describe a pipeline to produce disease survival subtypes from the output of our proposed method (Figure 2). The three pipeline inputs include: (i) paired-sample isoform measurements, (ii) a database of pathway annotations, and (iii) survival data. First, odds ratios and locFDR values are calculated using N1PAS (see Figure 2A) for the *N* patients in the data set (Table 1). Next, the odds ratios across all *P* pathways in the database are aggregated into an *N × P* matrix (Figure 2B). To reduce noise from non-informative pathway signal, the pathways are filtered to only pathways in which at least one patient is significantly enriched with ASGs (FDR_loc_ *<* 20%). This produces a new odds-ratio matrix (Figure 2C) with P′pathways (*P* minus the number of filtered pathways). Now, patients are clustered (unsupervised) into two groups (for simplicity and potential clinical utility) using only the odds ratios for a single pathway (Figure 2D). To this end, we used partitioning around medoids, a robust version of k-means with the number of groups *a priori* set to two. For each clustering (one per pathway), Kaplain–Meier estimates (Klein and Moeschberger, 1997) of the survival curves are computed (Figure 2E). A log-rank *p*-value assesses whether these two curves are distinct. Moreover, we devise the construction of an empirical null distribution of log-rank *p*-values for a pathway (Figure 2F). This procedure is similar to the *empirical p-value* approach above. We begin with shuffling patient’s odds ratios across the P′pathways. Thus, this forms a synthetic *null pathway* with values not tied to any particular pathway annotation while preserving the patient distributional characteristics. The patients were then clustered using these null values, and survival log-rank *p*-values were computed. This process was repeated 2000 times to produce an empirical null distribution of odds ratios. The observed log-rank *p*-value for each pathway is compared to this null distribution (red line in Figure 2F). This results in an empirical *p*-value for every pathway under consideration. Then the empirical *p*-values are adjusted using a Benjamini–Hochberg FDR adjustment (FDR_BH_). Pathways withFDR_BH_ < 20% are identified as survival-relevant pathways (Figure 2G). Clusters resulting using the N1PAS metrics within relevant pathways provide disease subtypes with distinct survival curves (Figure 2H) with the added benefit of simple diagnostic tools – relevant pathway odds ratio thresholds as depicted in Figure 2I.

### Alternative Approaches to Survival Subtyping

To explore whether pairing and aggregating alternative spliced genes at the pathway level improves the detection of survival-relevant pathways, we modify the above pipeline by systematically modifying the input to the binary clustering in three ways: (1) use only the tumor isoform expression (as opposed to N1PAS odds ratios and locFDR), (2) use the difference in tumor isoform expression from the normal isoform expression, and (3) use the Hellinger distances corresponding to each of the 4133 KEGG-associated genes The first method differs from N1PAS in that for each pathway *W* with *I_W_* associated isoforms (all isoforms relating to the *G* genes annotated *W*), all *I_W_* isoform measurements are included in the call to the PAM clustering algorithm. The second method uses both the tumor and normal isoform expression, but is only concerned with differential expression not alternative splicing patterns. The third method quantifies differential isoform usage, but does not aggregate these patterns at the pathway level (as in N1PAS). As we defined the Hellinger distance only when both samples expressed the gene, care was needed to form a complete 51 × 4133 matrix of Hellinger distances. Specifically, any missing gene-wise distances were imputed with a patient’s mean Hellinger distance across all genes. Each method produces two patient clusters as in Figure 2D and survival curves are fit for each of the 206 pathways. To construct an *empirical null* of survival *p*-values, each patient’s *I_W_* isoforms or genes were shuffled to disrupt correspondence with the specified pathway’s true annotations (similar to the other permutated distributions above) and two clusters are determined. Relevant pathways are identified using the FDR-corrected empirical *p*-values as shown in Figure 2G.

### Simulation Study of N1PAS Empirical Operating Characteristics

To investigate the performance of our N1PAS methodology in practice, we conducted a series of Monte Carlo evaluations. We examined how two different inputs affect the test’s operating characteristics: (1) number of expressed genes, *G*, in the pathway and (2) proportion, π, of ASGs within the pathway over the background percentage of ASGs. We study the empirical false positive rates and (statistical) power to detect an enriched pathway based on permutations of patient-specific Hellinger distances for all 246 TCGA patients (Table 1) while varying the two above inputs. Our focus lies in providing practitioners guidance to calibrate what effect size N1PAS can reliably detect. The pathway odds ratio serves as an effect size in N1PAS. This effect size corresponds to the proportion of ASGs within a pathway (π), relative to the background level of ASGs. We explain the details of the simulation below.

To avoid over-simplistic parametric and statistical assumptions (e.g., independent isoform counts) and to anchor simulation results to our studied setting, we restricted our Monte Carlo experiments to permutated TCGA patient-specific Hellinger distances for genes annotated to KEGG pathways. First, Hellinger distances within each gene were computed for all 246 patients across the 6 TCGA data sets. This produces patient-specific distributions of distances that are then used to classify genes into two groups: ASG or not (as in Figure 1B) for each patient. The proportion of ASGs across all genes is what we refer to as the patient-specific *background level* and denote this quantity asπ_all_. Next, for each patient, we shuffle the gene labels to approximate a ‘null’ distribution of Hellinger distances as the values do not aggregate meaningfully into pathways. Then the number of expressed genes *G* (approximate gene set size) is selected from the set {15, 30, 50, 100} and an effect size π is selected from the set {0, 0.05, 0.10, 0.15, 0.20}. Next, we randomly select one of the 206 KEGG pathways that has at least *G* expressed genes (but not more than 5 genes larger than the *G*, to give an approximate gene set size) to label Hellinger distances with specific gene labels to induce the effect size π. Call this selected pathway the *specified pathway*. To induce the specified effect size, G^∗^(π + π_all_) gene labels within the specified pathway were randomly chosen and assigned randomly sampled Hellinger distances from the ASG group. The remaining genes were randomly assigned values from the non-ASG group. Then N1PAS was run on the permuted, modified Hellinger distance data for all 206 KEGG pathways. This process was completed 100 times across the 4 × 5 simulation configurations for a total of 2000 runs per patient. This results in 246 patients × 2000 runs per patient for a total of 492,000 simulated N1PAS runs.

## Results

This section describes observations from the target KEGG pathway validation study across six TCGA data sets (Table 1), an application of the disease-subtyping pipeline to the TCGA LUSC data set of 51 lung squamous cell carcinoma patients, and the Monte Carlo studies. Throughout, we focus on the interpretation of single-subject results to showcase the unique insights made possible by N1PAS. Overall, the results highlight the vast heterogeneity of splicing dysregulation among cancer patients, despite having the same disease.

### Target KEGG Pathway Validation Study

The frequency of significantly enriched pathways of ASGs varies greatly from data set to data set and patient to patient. Table 2 compiles the results of the KEGG target pathway validation study. We only scored pathways with at least 15 *expressed* genes (in either sample from a patient). Of the 206 KEGG, the number of pathways scored across all data sets was fairly constant (with a median of approximately 185 pathways). The locFDR significance threshold appears somewhat severe with the median percent significantly enriched ranging from 3 to 7.8% across data sets. *So, it is quite difficult in this small ontology to call a pathway as enriched with ASGs.* One should keep in mind that pathway can have a high proportion of ASGs yet still not be significantly enriched, especially if alternative splicing is rampant through the entire transcriptome.

**Table 2:** Summary for target capture validation study and empirical assessment.

TCGA (N)	Target KEGG pathway	Median # scored pathways	Median # of hit pathways	Target pathway capture % (*p*-value)	Median target pathway rank	Cancer pathway capture % (*p*-value)
LUSC (51)	**Non-small cell lung cancer**	186	11	4% (ns)	58	47% (*p* = 0.03)
LUAD (58)	**Non-small cell lung cancer**	183	10	9% (ns)	49	52% (*p* = 0.001)
PRAD (52)	**Prostate cancer**	185	6	15% (*p* < 0.001)	28	42% (*p* < 0.001)
THCA (59)	**Thyroid cancer**	185	9	20% (*p* < 0.001)	37	39% (ns)
UCEC (7)	**Endometrial cancer**	182	14	9% (*p* < 0.038)	44	57% (ns)
BLCA (19)	**Bladder cancer**	181	11	47% (*p* < 0.001)	11	68% (*p* = 0.004)

*206 KEGG pathways were used as the input. There is one target pathway per data set and nine cancer pathways in the KEGG ontology (9/206 = 0.044). A “pathway capture rate” is the proportion of patients with the target pathway (or set of pathways) found significantly enriched at FDRloc < 20% and odds ratio > 1. A “cancer pathway capture rate” is the proportion of patients with at least one of the nine cancer pathways that was found to be enriched. The “null pathway” capture is the rate of a randomly-constructed “pathway” being enriched and, similarly, the null “cancer pathway” is the rate of at least one of nine null pathways found enriched. See section Methods for Computational Experiments for details. The “median # scored pathways” contains the median of the distribution of the number of scored pathways across patients (as the pathway must have at least 15 expressed genes, this varies from patient to patient). Similarly, the “median target pathway rank” contains the median rank across the patients for the corresponding target pathway. ns, not significant.*

The target pathway capture rate (i.e., the proportion of patients with a significantly enriched target pathway) is greater than expected for most data sets. The KEGG pathway *bladder cancer* was the most often-captured target pathway, with 9 of the 19 (47%) Bladder Cancer (BLCA) patients significantly enriched. To put this in context, a random null pathway for the BLCA data was never captured at such a high rate in 2000 empirically-derived pathways (Table 2; *p* < 0.001). In fact, the target capture rate is greater than expected for all data sets except for the two lung cancer data sets (LUSC and LUAD). Moreover, the rate of cancer pathway capture is higher than expected from four of the six data sets.

**FIGURE 3:**
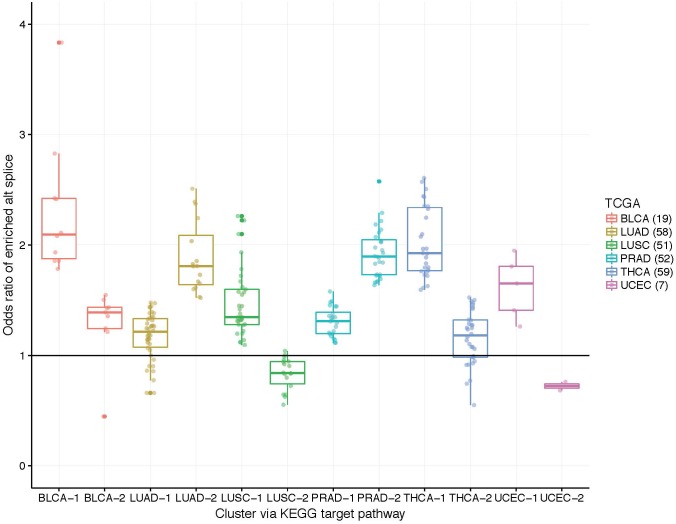
Boxplots of patient-specific odds ratios (OR) of the target KEGG pathway for the six TCGA data sets. Patients (indicated by points) were clustered using only the OR of the target pathway into two groups to unveil transcriptional response subtypes. Note that some patients exhibit less alternatively spliced genes within the target pathway than the background transcriptome (see LUAD, LUSC, and UCEC data sets; OR < 1).

While the capture rates on the surface appear lukewarm, we view the results as unveiling the need for subject-specific metrics. If one relied on cohort-based methods, the heterogeneity in splicing patterns would be missed. To explore this notion, we clustered patients into two groups using the odds ratios within target pathway. Figure 3 illustrates these grouping across the six data sets. Many patients show an enrichment of ASGs within patients (OR > 1) while others do not. Perhaps some patient’s disease mechanisms are not in a well-studied pathway and could be helped by innovative strategies.

### Applying the Disease-Subtyping Pipeline to the Non-small Cell Lung Cancer (LUSC) Data Set

**FIGURE 4:**
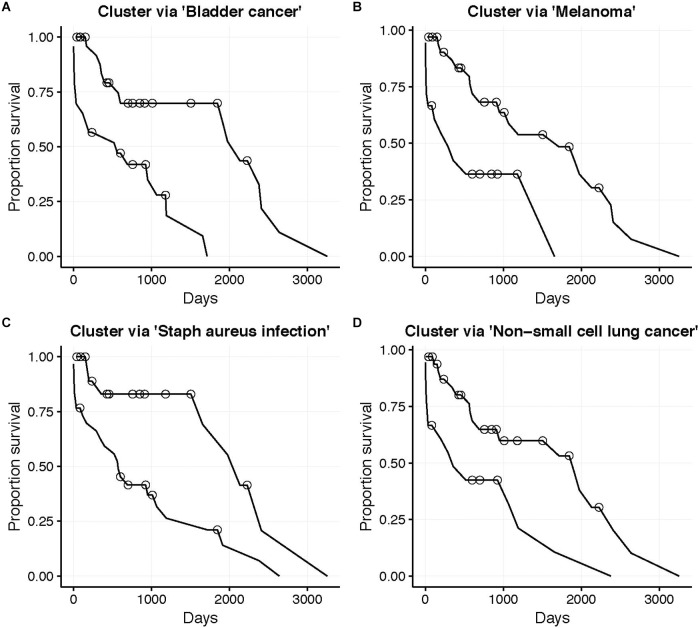
LUSC patient (*N* = 51, 32 deaths) survival curves. Subgroups determined by unsupervised clustering into two groups (*Better* and *Worse*) using the odds ratio for the corresponding KEGG pathway. **(A)** Bladder cancer pathway (N_Better_ = 28), **(B)** Melanoma pathway (N_Better_ = 33), **(C)** Staphylococcus aureus infection pathway (N_Better_ = 21), and **(D)** Non-small cell lung cancer pathway (N_Better_ = 33). Circles indicate censorship. All log-rank *p* < 0.01.

The low target-capture rate (3%) and high cancer pathway capture rate (47%) for the LUSC data set (Table 3, first row) and apparent *impoverishment* of ASGs for some patients (Figure 3) presents an interesting dilemma. Is the target pathway useful for these data? Or are there other, more interesting pathways related to patient outcomes? For this quandary, we applied the survival-subtyping pipeline to the 51 LUSC paired normal-tumor isoform expression, using KEGG pathways, and clinical survival data (32 deaths observed). Following the pipeline workflow illustrated in Figure 2, the 206 KEGG pathways meeting the filtering criteria were scored for each of the 51 patients. Next, the odds ratios were aggregated into an *N × P* matrix (Figure 2B). *P* is determined by the number of pathways scored in at least one patient. For these data, there are 174 such pathways. The pathways were further filtered to reduce noise to include only the 101 pathways found significantly enriched in at least one patient (Figure 2C). Next, each pathway is assessed one at a time to determine if unsupervised clustering of patients, based on odds ratios, into disease subtypes produces distinct survival curves (Figure 2D–G). Table 3 displays the discovered *survival-relevant pathways* (Diaz et al., 2017). Survival curves for the top-four pathways are provided in Figure 4.

**Table 3:** Non-small cell lung cancer (LUSC) pathways selected by subtyping pipeline.

Rank	KEGG description	Log-rank *p*-value	Empirical *p*-value (2000 reps)	# genes	% significant enrichments	% genes shared w/ target pathway
1	Bladder cancer	0.0001	0.0005	42	18%	52%
2	Melanoma	0.002	0.003	71	24%	47%
3	Staphylococcus aureus infection	0.003	0.004	55	18%	0%
**4**	**Non-small cell lung cancer^∗^**	**0.005**	**0.007**	**54**	**4%**	**100%**
5	Renal cell carcinoma	0.006	0.009	70	18%	36%

*Top-five ranked pathways out of 101 KEGG pathways enriched for at least one of the 51 patients (FDR < 20%). Pathways are ranked by log-rank p-value between survival curves corresponding to clustering of enrichment odds ratios. As shown by the high prioritization of the biologically relevant LUSC target pathway in this table, the proposed method using ORs of spliced pathways outperforms alternate analytical processing of alternative splicing signal as input to clustering as shown in Table 5. The columns headings explained: The “log-rank p-value” corresponds to the standard Kaplan-Meier p-value (unadjusted) for the pathway-based clustering. The “empirical p-value” results from our permutation test. The “# genes” is the number of annotated genes from KEGG. “% significant enrichments” column contains the percentage of subjects that were identified as having the significant enrichment of ASGs. The “% genes shared w/target pathway” contains the percent of genes annotated to the target pathway for each top-rank pathway ^∗^ = Target pathway. Bolded values denote the summary statistics of the LUSC target pathway.*

Thus, the N1PAS odds ratios and locFDR values were able to predict survival in the LUSC data set, as five pathways were found atFDR_BH_ < 20% (Table 3). Interestingly, four of these pathways relate to cancer with the fourth-ranked pathway being the LUSC *target pathway*. Surprisingly, the third-ranked pathway is the *staphylococcus aureus infection* KEGG pathway, which may present an orthogonal explanation for a poor survival outcome.

**FIGURE 5:**
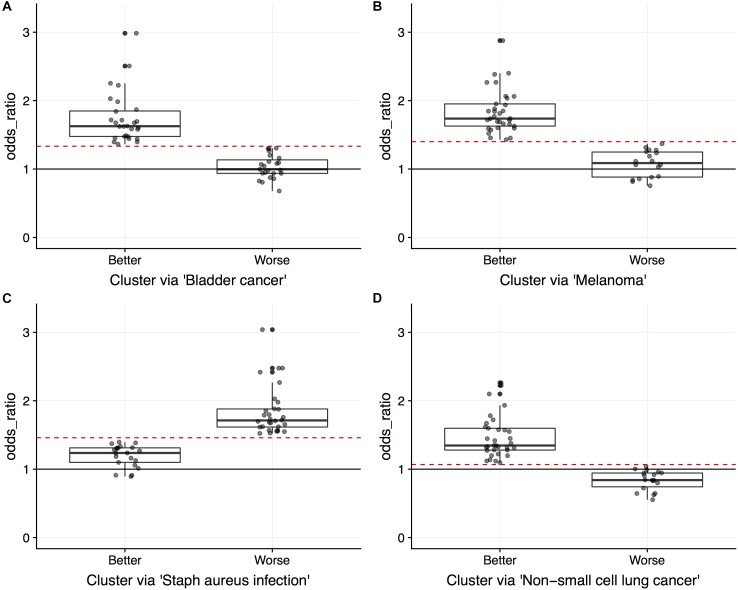
Boxplots overlaid with points indicating the odds ratios of pathway enriched with ASGs. Points indicate values for the 51 LUSC patients, split by unsupervised cluster assignment into two groups (“Better” and “Worse” as interpreted by survival data) for pathways **(A)** “Bladder cancer” (N_Better_ = 28), **(B)** “Melanoma” (N_Better_ = 33), **(C)** “Staphylococcus aureus infection” (N_Better_ = 21), and **(D)** “Non-small cell lung cancer” (N_Better_ = 33). Circles indicate censorship. Since the clustering is done on a single vector of odds ratios for each pathway, an odds ratio threshold can separate the patients (red dashed lines; midpoint between clusters).

Figure 5 displays the odds ratio distribution within the top-four relevant pathways split by patient clusters (subtypes). Patient subtypes have been annotated as *Better* or *Worse* based on inspection of the survival curves. For the three cancer-annotated pathways, a higher odds ratio of enrichment with ASGs is associated with a *better* survival outcome. This paradoxical observation may be the result of drug efficiency directed at known cancer biology. The reverse pattern lies in the staphylococcus-related pathway – more abundant alternative splicing in the pathway results in poor survival. Simple diagnostic rules can be found by inspection of the boxplots in Figure 5. For example, a patient-specific odds ratio less than 1 in the *non-small cell lung cancer pathway* (*target pathway*; Figure 5D) indicates a poorer prognosis.

One practical issue with subtyping a patient using multiple survival-relevant pathways is that different clusterings may disagree with prognosis (*Better* using one pathway’s odd ratios and *Worse* using another’s). This will require care to uncouple for the patient at hand. For example, a patient may exhibit a worse prognosis in the *staphylococcus aureus infection* pathway, but a *better* prognosis in the cancer-annotated pathways. Hypothetically, this patient may then respond well to the standard treatment in combination with an innovative treatment to address the dysregulation in the *staphylococcus* pathway.

To gain insight into subtype overlap within our LUSC case study, we explore agreement across the top-four survival-relevant pathways (Table 3). To quantify the agreement, we compute the Jaccard index asJ_1,2_ = $\frac{\left|{\text{G}}_{1}\cap {\text{G}}_{2}\right|}{{\text{|G}}_{1}\cup {\text{G}}_{2}|}$, where *G*_1_, *G*_2_ are the sets of patients clustered using the 1st and 2nd top pathways (for either the *Better* or *Worse* subtype). Table 4 displays the Jaccard indices for all pairs of top ranked pathways for both subtypes. The quantities suggest that agreement is stronger for the *Better* subtype (average Jaccard index of 0.4942, compared to the average *Worse* index of 0.3255). This is interesting as it may indicate that individual lung cancer patients display unique dysregulated pathways (motivating precise treatments). We also observe that subtyping based on the three cancer-associated pathways (ranks 1, 2, 4) generally agree well for the *Better* patients. But these *Better* subtypes from cancer pathways agree poorly with the *staphylococcus aureus infection* subtyping. The *Worse* subtypes generally agree less well than the *Better* subtypes, and with a similar trend in disagreement with the *staphylococcus* pathway. This suggests a distinct survival-related signal in this pathway from the cancer-annotated dysregulation. Of course, conflicting subtypes will complicate clinical application and slow any decision process, thus limiting our proposed approach.

**Table 4:** Subtype agreement across the top-four LUSC survival-relevant pathways.

Subtype	J_12_	J_13_	J_14_	J_23_	J_24_	J_34_
Better	0.605	0.289	0.605	0.370	0.784	0.313
Worse	0.464	0.159	0.464	0.147	0.636	0.083

*The Jaccard index measures the proportion of overlapping patients selected in either the Better or Worse group by each pair of top-ranked pathways (Table 3). The first, second, and fourth pathways are all associated with cancer annotated genes and their comparisons show highest agreement scores.*

### Comparing Alternative Approaches to Survival Subtyping

As detailed in section Alternative Approaches to Survival Subtyping, the input data to the clustering step of our proposed subtyping pipeline was modified using three straightforward alternative approaches for the 51 LUSC patients: (1) using tumor isoform expression, (2) using the difference in isoform expression between tumor and normal samples, and (3) Hellinger distance data for the 4133 genes annotated to KEGG pathways. Table 5 summaries the pathway rankings and statistical significance of survival prediction for the three alternative approaches. We’ll discuss the results of each method in turn.

**Table 5:** Complementary study of LUSC survival-relevant pathway rankings by subtyping pipeline using three alternative analytical transformations (first column; clustering inputs) before clustering.

Clustering input	Rank	KEGG description	Log-rank *p*-value	Empirical *p*-value	Empirical FDR_BH_
Tumor isoform expression only	1	Phenylalanine metabolism	0.0017	0.005	0.515
	2	Complement and coagulation cascades	0.0024	0.0030	0.515
	3	Pentose phosphate pathway	0.0106	0.0245	0.750
	4	Vasopressin-regulated water reabsorption	0.0112	0.018	0.750
	…	…	…	…	…
	**64**	Non-small cell lung cancer (target pathway)	0.130	0.217	0.750
Difference in tumor and normal isoform expression	1	Phenylalanine metabolism	0.0014	0.003	0.618
	2	RNA degradation	0.0034	0.0140	0.747
	3	GnRH signaling pathway	0.0037	0.017	0.747
	4	Glycolysis/Gluconeogenesis	0.0130	0.0285	0.747
	…	…	…	…	…
	**144**	Non-small cell lung cancer (target pathway)	0.571	0.6025	0.854
Hellinger distance within genes across isoform expression	1	Chronic myeloid leukemia	< 0.001	0.0005	0.103
	2	Osteoclast differentiation	0.0035	0.0060	0.618
	3	Steroid biosynthesis	0.0111	0.0150	0.762
	4	Progesterone-mediated oocyte maturation	0.0130	0.0155	0.762
	…	…	…	…	…
	**30**	Non-small cell lung cancer (target pathway)	0.1186	0.136	0.899

*In order to demonstrate that the proposed method evaluated in Table 3 outperforms straightforward analytical transformations (first column, clustering inputs), we conducted these additional substudies (section Methods for Computational Experiments). The non-small cell lung cancer pathways of KEGG is the most relevant to LUSC survival and is ranked higher (#4, Table 3) in the proposed method than in the alternate ones presented here. Ranked pathways of the 206 KEGG pathways with at least 15 and no more than 500 genes. Empirical p-values are constructed by comparing to survival curves generated from clustering on permutations of the clustering input data expression. Bolded values denote the rank of the LUSC target pathway.*

The tumor-expression method clusters solely on the isoform expression and uses no dimension reduction techniques prior to clustering. This could perhaps provide more information in the cluster assignments. No pathways, however, were found to be statistically significant at FDR < 50%. But this approach uses only half of the expression data input into N1PAS; thus, it may suffer reduced ability to detect survival-relevant pathways based on that fact alone. The top pathways’ descriptions appear to be less relevant than the results for N1PAS (Table 3). The *non-small cell lung carcinoma* target pathway is ranked relatively poorly (64 out of 206 pathways, compared to the fourth-ranked pathway using N1PAS in Table 3). These results seem to imply that clustering on the dynamic, individualized metrics of differential isoform usage within pathways provides higher resolution for survival prediction than tumor isoform expression alone.

Next, we use the difference in tumor and normal isoform expression as input to the binary clustering of LUSC patients. The top-ranked survival-relevant pathway, *phenylalanine metabolism*, agrees with the tumor-only results. The other top pathways disagree, suggesting a distinct signal. The *non-small cell lung carcinoma* target pathway’s rank dropped substantially in this approach to a rank of 144 out of 206 KEGG pathways when compared to the N1PAS results reported in Table 3. No pathways were found to significantly produce separate survival curves at FDR < 50%.

Finally, we use the Hellinger distances for each gene across the 51 LUSC patients to cluster patients. This signal is somewhat nearer to N1PAS and is concerned with alternative splicing patterns. N1PAS takes these distances one step further by aggregating the signal into pathway-level enrichment of ASGs (by operating with the odds ratios; Figure 1C). The *non-small cell lung carcinoma* target pathway rank is also low (30 out of 206 KEGG pathways) when compared to the N1PAS method reported in Table 3. The top hit pathway, *chronic myeloid leukemia*, was significant at FDR < 20% – in contrast to the two other alternative methods.

Put together, we see that the use of N1PAS odds ratios and significance assessment increases the ability to find survival-relevant subtypes within pathways. The results also suggest that alternative splicing analyses within pathways present a complementary viewpoint to expression-based workflows.

### Simulation Results

Our simulation results provide insight into N1PAS empirical operating characteristics, specifically simulated false-positive rates and statistical power. Among the 492,000 N1PAS runs, there were 625 algorithm failures (0.127%) due to misfit odds ratio mixture modeling. This may happen in practice, although rarely, and the parameters of locFDR will have to be manually adjusted. Here, we discarded these runs from the results. Each patient displays a different Hellinger distance distribution with their own background level of ASGs (π_all_). The average π_all_ across the 246 patients is 0.1914, but vary from 0.1088 to 0.4417 with the middle 50% of the rates vary from 0.1613 to 0.2122.

**FIGURE 6:**
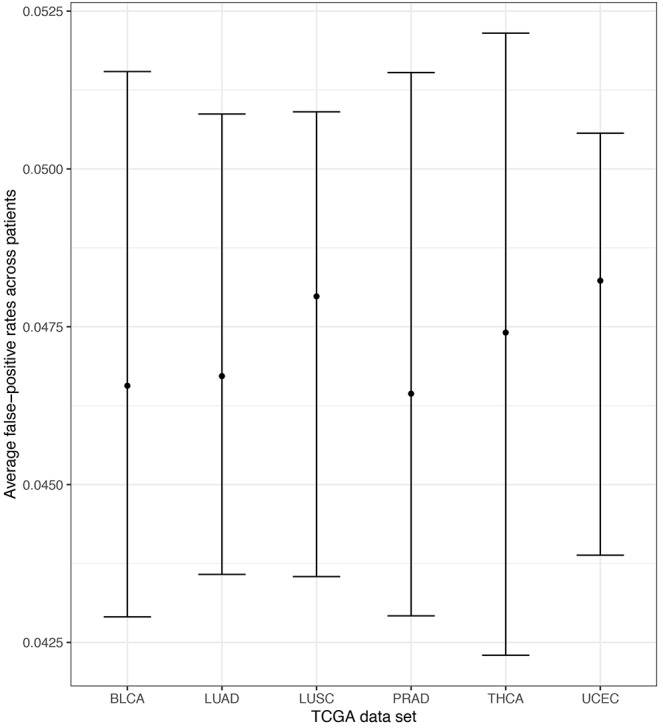
Extensive simulations of false-positive rates under the assumption of locFDR < 20% shows conservative 4–5.25% false-positive rates for the N1PAS method. Empirical false-positive rates are represented as dots for each TCGA data set, based on simulated patient-specific Hellinger distance data. Results reported for π = 0 (see text). Horizontal bars are 95% confidence intervals around the overall mean false-positive rate (across patients within the data set). For example, the confidence interval for LUSC was constructed by first finding the average patient-specific false-positive rate for all 51 patients (51 patients × 100 replicates/patient = 5100 simulations for LUSC). Then, the standard error of the overall mean was estimated using the sample standard deviation across patients and a normal approximation is used to determine the confidence bounds.

Empirical false-positive error rates correspond to the π = 0 simulation setting (no pathway is specified to have an enrichment of ASGs). ASGs can aggregate in pathways by chance in this setting. We calculated the simulated false-positive rate as the number of detected pathways among the 206 KEGG pathways under permuted patient-specific Hellinger distances. We performed 100 simulation replicates per patient and computed the patient-specific average false-positive rates. Further, we pooled these mean rates within each TCGA data set and computed the empirical standard error of the mean to assess variability across patients. Finally, we computed pointwise 95% confidence intervals for the data set mean false-positive rate using a normal approximation centered at the overall mean with the observed standard error. Figure 6 displays these mean false-positive rate estimates. The patient-specific average false-positive rates vary tightly around 0.04–0.0525 with centers between 0.045 and 0.050. This is interesting since the decision threshold used in N1PAS was locFDR > 0.20, and we observe rates much lower than this specification. This trend in false-positive rates persists across TCGA data sets. In sum, the simulated false-positive rate data suggests adequate (if not conservative) method performance with respect to false discovery rates.

**FIGURE 7:**
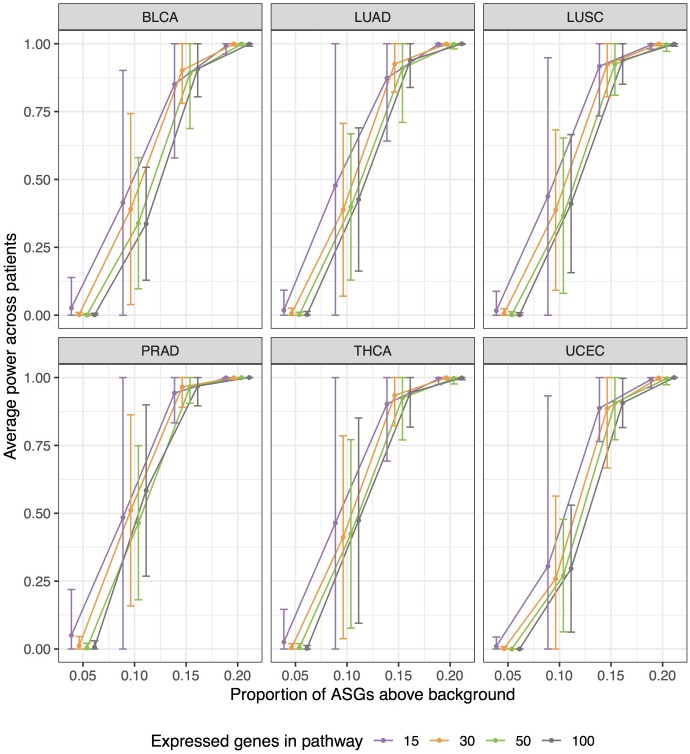
Extensive simulations show substantial statistical power of N1PAS method in detecting pathways enriched with Alternatively Spliced Genes (ASGs) when the specified pathway’s proportion (π) is 15% higher than subject-specific background level of ASGs (π_all_). Approximately 400,000 simulations were conducted to assess power by varying expressed genes in the specified pathway (*G*), proportions π, and gene labels assignment for patient-specific Hellinger distances. Empirical power estimates are displayed as jittered dots for each TCGA data set, based on the detection rate of a specified pathway. Horizontal bars are 95% confidence intervals around the overall mean power (across patients within the data set, stratified by *G* and *π*). Power curves were constructed using linear interpolation between pointwise power estimates (dots) at fixed values of simulation configurations.

Empirical power estimates, as detection rates, from our simulations are graphed in Figure 7. Power calculations correspond to simulations withπ > 0. Here we call *power* the observed detection rate of a specified pathway, with an induced enrichment of ASGs. Figure 7 presents the results as a function of the ASG proportion above background (π), and stratifies power curves by expressed genes in pathway (*G*). We increment the effect size π from 0.05 to 0.20 to calibrate how sensitively N1PAS can detect an enriched pathway. 95% confidence intervals for mean power were constructed at each pair (π *G*) within each data set as above in the false-positive rate study.

Patterns in Figure 7 show a trend toward increasing power while increasing the effect size π. N1PAS rarely detects a 5% enrichment of ASGs over the background level. At 10%, however, detection is possible but highly variable. At 15% ASGs above background, power becomes more reliable with 75% of average power estimates above 0.9. At 20%, the specified pathway in almost always detected (minimum patient-specific power = 0.9916). The trends in simulated power are generally consistent across TCGA data sets at extreme values of π, 0 or 0.2. There are, however, some differences in central tendency and variability across TCGA data sets atπ = 0.1, 0.15. For example, PRAD patients show more power to detect a specified pathway with a 15% ASG enrichment than BLCA. As one may expect, power estimates are more variable with a smaller number of expressed genes *G* for immediate values of π (e.g., 0.1 or 0.15).

In general, these results suggest that N1PAS procedure exhibits good false-positive error control and excellent power, at least under the settings chosen for these simulations.

## Discussion

This study creates a first look at personalized alternatively splicing patterns within pathways. As such, these patterns are likely complementary to other ‘Omics measures, and thus the proposed N1PAS strategy could become an additional tool to bridge the gap between RNA-Seq data and clinical translation. For example, one could investigate whether splicing events occur in a coordinated way in response to a stimulus or perturbation like a medication. A practical limitation is the relative rarity of paired RNA-Seq data (compared to single-sample expression data). It’s true that paired unaffected and cancer tissues are currently uncommon. Yet novel experimental designs and corresponding analyses will drive data collection protocols. Indeed, as the National Institute of Health and National Cancer Institute announced (Collins and Varmus, 2015), there is interest in promoting and developing creative new assays and analytics for predicting individualized disease mechanisms and treatments. The N1PAS methods is a proof of concept that demonstrates, with the blessing of high dimensionality and integration of external knowledge, that signal can aggregate within gene sets of paired samples and improve their mechanistic interpretation.

To make clear of a potential clinical use of N1PAS within the survival subtyping pipeline of Omics signals, imagine the following scenario: a patient suffering from non-small cell lung cancer consents to paired tumor-normal RNA-sequencing. Patient-specific N1PAS odds ratios are first computed in the five survival-relevant pathways. Each survival-relevant pathway has an associated odds ratio threshold (red dashed lines in Figure 5). Based on this threshold, the patient is stratified into either the *Better* or *Worse* survival groups. This informs on both prognosis and on patient-specific disease mechanisms. Our proposed method, however, only considers one pathway at a time and therefore only partially explains survival, compared to using all the odds ratios. Future studies could improve survival prediction through aggregation of pathway metrics and potentially have greater clinical utility in terms of prognosis in addition to combining multiple ‘Omics measurements.

There are, of course, limitations and caveats to the methodology. The proportion clustering approach seeks to quantify differential relative isoform usage and not differential *gene* expression. However, a gene could be differentially expressed based on the magnitudes of the sum across isoforms (typical gene expression) as well as exhibit differential isoform usage. As such, the signal obtained by N1PAS may not be purely alternative splicing as traditional DEGs may still contribute. Another issue is that different isoforms may not always be indicative of differential protein structure or activity and the biological impact may be minimal in these situations. The model does not currently account for any noise that may be present in RNA-Seq measurements, an important consideration in the N-of-1 setting. Along those lines, the estimated proportions *p*_gA_ in Equation (1) depend on the initial accuracy of the isoform abundances. This accuracy depends on several aspects such as read depth, number of expressed isoforms, and the specific method that has been used for estimating the transcript abundance. Therefore, the estimated proportions may have different variances and other statistical properties. Our method does not explicitly account for these differences. An important extension of our model could include this uncertainty in estimating the proportions for a more holistic and realistic formulation.

We acknowledge some limitations to the computational experiments in the study. The significance of the survival curves has been assessed on a single retrospective dataset and could be over-optimistic. Future studies should include prospective independent datasets. Our choice of pathway database is outdated and more current databases may provide more informed discoveries. One could imagine a variety of alternative methods to form comparisons against N1PAS. For example, *p*-value aggregation methods (such as Fisher’s method) could be explored. Care must be taken, however, as these methods often assume independent measurements. Future studies could develop more sophisticated and novel *p*-value aggregation approaches in this setting. Additionally, it would be interesting to vary the pathway definitions across several databases in future studies. Lastly, this proof of concept study was designed to demonstrate the utility of alternative splicing signals in absence of other signal to avoid confounders. Therefore, in future studies, this method should be opportunistically combined with any ‘Omics as well as clinical signals in order to translate clinically useful predictions with high accuracy.

## Conclusion

We proposed a single-subject methodology, N-of-1-*pathways* Alternatively Spliced, to quantify differential mRNA isoform usage within biological pathways from paired-sample RNA-Seq data. A target pathway validation study on paired normal-tumor samples from TCGA reveals that in most data sets the identified pathways generally concur with the annotated disease (*bladder cancer* pathway was more likely to be enriched with ASGs). More than just providing validation, this study also highlights the vast patient-to-patient heterogeneity in alternative splicing patterns. This heterogeneity actually motivates our N-of-1 approach: despite having the same disease, patients vary greatly in their splicing dysregulation. Our identification of subject-specific splicing dysregulation offers targets for personalized interventions and monitoring plans. Next, we applied our N1PAS single-subject metrics to predict survival within a novel subtyping pipeline. The output of this pipeline contains easy-to-interpret diagnostics to enable precision medicine from transcriptome data. Finally, we showed adequate statistical power and false-positive rates in simulation studies.

## Author Contributions

AS and YA convinced the foundational concepts, designed experiments, and explored the results. AS developed the methodology and drafted the manuscript. AS and DA conducted the computational experiments. YA contributed sections. AS and CK developed the figures. All authors contributed to manuscript revision, read and approved the submitted version.

## Code Availability

All code used to conduct this study are freely available at https://github.com/grizant/nof1-splice. An R package to conduct N1PAS, including vignettes, is freely available at https://github.com/grizant/n1pas/tree/master.

## Conflict of Interest Statement

The authors declare that the research was conducted in the absence of any commercial or financial relationships that could be construed as a potential conflict of interest.

## Footnotes

1 http://gdac.broadinstitute.org/
